# Comparative transcriptomics of mountain pine beetle pheromone-biosynthetic tissues and functional analysis of CYP6DE3

**DOI:** 10.1186/s12864-017-3696-4

**Published:** 2017-04-20

**Authors:** J. A. Nadeau, J. Petereit, R. L. Tillett, K. Jung, M. Fotoohi, M. MacLean, S. Young, K. Schlauch, G. J. Blomquist, C. Tittiger

**Affiliations:** 1 Department of Biochemistry and Molecular Biology, University of Nevada, Reno, NV 89557 USA; 2 Biomedical Engineering Department, University of Nevada, Reno, NV 89557 USA; 3 Nevada INBRE Bioinformatics Core, University of Nevada, Reno, NV 89557 USA

**Keywords:** Transcriptomics, RNA-Seq, Bark beetle, *Dendroctonus*, Pheromone, P450

## Abstract

**Background:**

The mountain pine beetle (MPB, *Dendroctonus ponderosae* Hopkins) is a highly destructive pest of pine forests in western North America. During flight to a new host tree and initiation of feeding, mountain pine beetles release aggregation pheromones. The biosynthetic pathways of these pheromones are sex-specific and localized in the midgut and fat body, but the enzymes involved have not all been identified or characterized.

**Results:**

We used a comparative RNA-Seq analysis between fed and unfed male and female MPB midguts and fat bodies to identify candidate genes involved in pheromone biosynthesis. The 13,407 potentially unique transcripts showed clear separation based on feeding state and gender. Gene co-expression network construction and examination using **petal** identified gene groups that were tightly connected. This, as well as other co-expression and gene ontology analyses, identified all four known pheromone biosynthetic genes, confirmed the tentative identification of four others from a previous study, and suggested nine novel candidates. One cytochrome P450 monooxygenase, CYP6DE3, identified as a possible *exo*-brevicomin-biosynthetic enzyme in this study, was functionally characterized and likely is involved in resin detoxification rather than pheromone biosynthesis.

**Conclusions:**

Our analysis supported previously characterized pheromone-biosynthetic genes involved in *exo*-brevicomin and frontalin biosynthesis and identified a number of candidate cytochrome P450 monooxygenases and a putative cyclase for further studies. Functional analyses of CYP6DE3 suggest its role in resin detoxification and underscore the limitation of using high-throughput data to tentatively identify candidate genes. Further functional analyses of candidate genes found in this study should lead to the full characterization of MPB pheromone biosynthetic pathways and the identification of molecular targets for possible pest management strategies.

**Electronic supplementary material:**

The online version of this article (doi:10.1186/s12864-017-3696-4) contains supplementary material, which is available to authorized users.

## Background

**Fig. 1:**
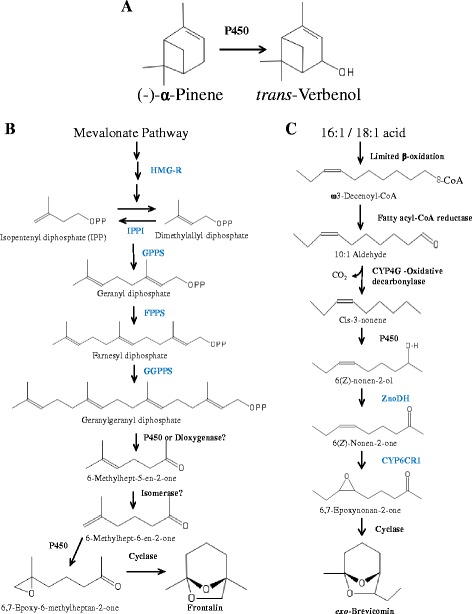
Pheromone-biosynthetic pathways. MPB pheromone biosynthetic pathways. **a** (–)-trans-Verbenol is produced by a single P450-mediated hydroxylation of (–)-α-pinene. **b** Frontalin biosynthesis occurs through the mevalonate pathway to geranylgeranyl diphosphate followed by multiple steps likely catalyzed by P450s, a dioxygenase, and a cyclase. **c** exo-Brevicomin production from long chain fatty acid precursors in the fat body of unfed males involves steps catalyzed by P450s, a short chain dehydrogenase, and a cyclase. Previously identified/characterized enzymes are in blue font. Chemical structures were generated using PubChem Sketcher V2.4, https://pubchem.ncbi.nlm.nih.gov/edit2/index.html

The mountain pine beetle (MPB) (*Dendroctonus ponderosae* Hopkins) uses three main pheromone components to coordinate the “mass attack” necessary to overcome a host tree’s defenses. Each component has a distinctive role and is produced from a different metabolic pathway (Fig. 1). Females produce the aggregation pheromone (–)-*trans*-verbenol [(1S,2R,5S)-4,6,6-trimethylbicyclo[3.1.1] hept-3-en-2-ol], to attract other males and females to a newly colonized host tree [1]. Biosynthesis of (–)-*trans*-verbenol likely requires cytochrome P450-mediated hydroxylation of the host tree produced monoterpene, (–)-α-pinene. Newly emerged males produce the aggregation pheromone *exo*-brevicomin [(1R,5S,7R)-7-ethyl-5-methyl-6,8-dioxabicyclo[3.2.1]octane] from long chain fatty acid precursors in the fat body [2, 3]. *exo*-Brevicomin production decreases substantially upon reaching the new host tree and mating [4]. The third major component, frontalin [(1R,5R)-1,5-dimethyl-6,8-dioxabicyclo[3.2.1]octane], is produced by feeding males and acts as an anti-aggregation signal to halt the attack and prevent overcrowding of the tree [4]. Frontalin biosynthesis uses the mevalonate pathway [5].

Various high-throughput studies, including a draft MPB genome [6], and various transcriptomic [7, 8] and proteomic [9] analyses have been directed at this highly destructive coniferous forest pest in order to better understand its physiology and to assist development of new management strategies. Aggregation and anti-aggregation pheromones in the MPB are synthesized in the midguts and fat bodies in a sex- and feeding status-specific manner [10]. Therefore, mRNAs encoding enzymes involved in pheromone-biosynthetic pathways may also be differentially expressed based on sex, feeding status, and tissue. Recently, Keeling et al. [11] reported transcriptome, proteome, and metabolome-level responses of unfed MPB treated with juvenile hormone (JH) III. JH III stimulates aggregation pheromone component production in bark beetles [5, 12–15], including frontalin and *trans*-verbenol in MPB, and several “pheromone-biosynthetic gene” candidates were identified by this study. However, the dynamics of the transcriptional response of JH III-treated insects can differ from those of fed insects [15–17] and the study noted that JH III treatment did not affect *exo*-brevicomin production. Thus, JH treatment may not induce differential expression of all genes involved in pheromone biosynthesis for the MPB.

Here, we report the results of an RNA-Seq study of MPB midguts and fat bodies based on sex and feeding status. We employed network, differential gene, and gene ontology enrichment analyses to isolate candidates involved in pheromone biosynthesis. Additionally, functional analysis of a candidate gene, *CYP6DE3*, identified by our RNA-Seq analysis for *exo*-brevicomin biosynthesis, indicates a role in resin detoxification rather than pheromone biosynthesis, reinforcing the need for functional assays to confirm possible roles of enzymes identified via bioinformatics approaches.

## Methods

### Tissue collection

Sections of mountain pine beetle-attacked lodgepole pine, *Pinus contorta*, were collected from Truckee, CA, USA, near the Northwoods Clubhouse in the Tahoe Donner housing subdivision (approx. N 39°20′37″ W 120°12′54″) on September 30, 2013. The beetles overwintered in the bolts and emerging adult beetles were collected and sexed as reported previously [3, 18]. For feeding experiments, fresh lodgepole pine bolts were obtained from the Whittell Forest, located in the Carson Range on the east slope of the Sierra Nevada (approx. N 39°16′29″ W 119°52′43″) in June, 2014 and stored at 4 °C prior to use. Females were fed by drilling small holes just beneath the bark, inserting the beetles head first, stapling a wire mesh over the occupied hole, incubating the bolt vertically for 24 h in the dark at room temperature and collecting the live beetles by stripping the bark. Fresh frass indicated that the beetles had fed. Males were fed using the same method except females were first placed head first into the holes for 24 h, followed by insertion of the males head first for 24 h and subsequent collection and sexing of the live beetles. Unfed beetles were incubated in 2 oz plastic cups with perforated lids in a dark drawer kept humid with small flasks filled with water and a paper towel for 24 h. All beetles were dissected following treatment to collect midgut and fat body tissue. Four replicates of pooled tissue from 10 beetles were collected for each of the four treatments (fed and unfed males and females) for a total of 16 samples. Midguts and fat bodies were immediately frozen in liquid nitrogen and stored at -80 °C for subsequent RNA extraction.

### RNA extraction and sequencing

Total RNA was isolated from the midguts and fat bodies pooled from 10 beetles per sample using an RNeasy Plant Mini Kit from Qiagen (Valencia, CA) and treated with RNase-Free DNase Set from Qiagen as described by the supplier’s manual. RNA was quantified using Quant-iT™ RiboGreen® reagent and a Labsystems Fluoroskan Ascent fluorescence plate reader. RNA integrity for each sample was determined using an Agilent 2100 Bioanalyzer and a Eukaryote Total RNA Nano Series II chip. Only samples with an RNA Integrity Number of eight or higher were used for sequencing [19]. RNA was precipitated and provided to the Georgia Genomics Facility (GGF) for library preparation and sequencing. GGF confirmed the quality of the total RNA using an Agilent 2100 Bioanalyzer, prepared barcoded cDNA libraries using a Kapa Stranded mRNA-Seq Kit (Wilmington, MA), and sequenced them on the Illumina NextSeq 500 using paired-end sequencing with a NextSeq 2x75 High Output Flow Cell.

### Sequence quality control

Sequence quality for each sample was characterized using FastQC (v. 0.11.2; http://www.bioinformatics.babraham.ac.uk/projects/fastqc/). Sequence pairs were trimmed and filtered for nucleotide-base quality and Illumina adapter sequences using Cutadapt v. 1.8.1 [20], with options set as follows: trimming of low-quality (phred quality ≤ 10) and “N” base calls from both ends of each read and removal of sequences with trimmed length < 35 nt.

### Sequence alignment and expression quantification

Before assembly, trimmed sequence pairs were compared to one another using the MaSuRCa ‘superreads.pl’ script, and if found to intersect (minimum k-mer 41), were combined into single “super-reads” [21]. Read pairs with no intersection were retained as separate paired-end reads. Single reads and read pairs were aligned to the Ensembl Metazoa (release-25; [22]) *D. ponderosae* reference genome [6] using the HISAT spliced read alignment tool (v. 0.1.6-beta; [23]). The coordinates of each known *Dendroctonus* gene and its exons were extracted from the Ensembl Metazoa Gene Transfer Format (GTF) file and supplied to HISAT at time of alignment (via HISAT’s ‘--known-splicesite-infile’ option). Resultant alignments were compressed from the sequence alignment/map (SAM) format to the binary BAM format [24]. Upon alignment, the raw counts of reads and read pairs aligned to each gene were totaled using the featureCounts tool of the subread package (v. 1.4.6-p1; [25]). Reads were counted once per pair and summarized for gene loci, with only read pairs aligned to a unique transcribed location included in the count totals.

Transcripts underwent two filtering steps. First, those with no counts for thirteen or more out of the sixteen (75%) samples were excluded. Then, all transcripts with less than 10 fragments (counts) observed in all sixteen samples were excluded. Data were normalized using the standard median ratio method for RNA-Seq data [26]. Principal component analysis (PCA) was performed on the normalized and filtered zero-centered counts per million data using singular value decomposition to validate clear separation between gender and feeding status of the biological replicates of MPBs.

### Annotation

*Dendroctonus ponderosae* gene descriptors and annotated Interpro protein domains [27] were obtained from Ensembl Metazoa, via the BioMart interface [28]. Interpro2GO file (v. 2016/03/19 11:04:26) was used to map Interpro IDs to gene ontology (GO) terms.

### Validation of RNA-Seq data by quantitative PCR

To validate RNA-Seq data, the transcript levels of 15 genes (Table 2) were examined by quantitative reverse transcriptase PCR (qRT-PCR). Genes were chosen by their notable differential expression between feeding states in male beetles. Aliquots consisting of approximately 500 ng of total RNA from a subset of samples (fed male replicate 4, unfed male replicate 4, fed female replicate 4, unfed female replicate 4, fed male replicate 1, unfed male replicate 3, unfed female replicate 2) were used to make cDNA using iScript™ Reverse Transcription Supermix (Bio Rad, Hercules, CA). PCR was conducted in a 20 μL reaction consisting of iTaq™ Universal SYBR® Green Supermix (Bio Rad) and 2 μL of template for 40 cycles of 95 °C for 5 s and 60 °C for 30 s on a Bio Rad CFX96 Real-Time PCR Machine (Bio Rad). Primers designed to amplify specific transcripts of these genes were designed using IDT Primer Quest and melt curves were produced to ensure primer specificity and proper PCR temperature cycling parameters (Additional file 1). For each cDNA sample the PCR reactions were conducted in triplicate and relative target gene expression was normalized to that of YQE_05788, which encodes ribosomal protein S3P. Ribosomal protein S3 is a relatively more stable normalizing gene for qRT-PCR in another beetle, *Tribolium castaneum*, compared to the more routinely used actin or tubulin genes [29]. Fold change was calculated for each normalized gene in relation to the expression of the unfed male treatment using the 2^-ΔΔCT^ method [30]. For each gene, Pearson and Spearman Correlation Coefficients were computed between the seven samples measured by qRT-PCR and RNA-Seq.

### Co-expression network

Gene co-expression networks are node-edge graphs. Nodes represent genes that are connected by edges if there is an association between genes as defined by a co-expression measure [31]. Structural components of co-expression networks are used to identify densely connected subgraph, called gene modules. Genes within a module share similar expression patterns, thus they are hypothesized to have similar gene function, to share pathway membership, or to be co-regulated. A co-expression network of the filtered and normalized counts per million of 11,342 mountain pine beetle genes was generated via **petal**, a co-expression network construction and analysis tool [32]. The entire dataset of 11,342 genes over 16 measures was loaded, along with a list of previously confirmed and hypothesized pheromone biosynthetic genes. No other input was specified.

### Differential gene analysis

Differential gene expression between the feeding conditions and the genders were examined using DESeq2 [26]. Four comparisons, male fed (MF) vs. female fed (FF), MF vs. male unfed (MU), MU vs. female unfed (FU) and FF vs. FU, were considered using simple contrasts. A multiple testing correction was performed for each of the four comparisons to adjust for the false discovery rate [33]. The two other possible comparisons (MU vs. FF and MF vs. FU) were not considered because they are less likely to inform regarding putative pheromone-biosynthetic genes. Genes with absolute value of the log_2_ fold change greater than one and an adjusted p-value less than 0.01 were retained for further analysis. Venn diagrams were prepared within R to visualize the intersection of the statistically-significant differentially-expressed genes between the considered comparisons (Additional file 2).

### Gene ontology enrichment analysis

Gene Ontology (GO) enrichment analyses of statistically-significant differentially-expressed gene groups were conducted to identify over-represented molecular functions and metabolic processes. BiNGO (v. 3.0.3) [34] within Cytoscape (v. 3.3.0) and GO file (v. 1.2 2016/03/01) were utilized. GO terms with adjusted significance values less than 0.05 upon a Benjamini-Hochberg adjustment [33] were considered for further investigation.

### Recombinant CYP6DE3 expression

RNA was extracted from two whole beetles using a RNeasy Plant Mini Kit (Qiagen) as per the manufacturer’s instructions. First strand cDNA was synthesized using Superscript III Reverse Transcriptase (Invitrogen, Carlsbad, CA) as per the manual. The CYP6DE3 open reading frame (ORF) was amplified by PCR using CYP6DE3F1 and CYP6DE3R1 primers (Additional file 1) and CloneAmp HiFi PCR Premix (Takara Bio USA, Inc., Mountain View, CA), cloned into pENTR4 modified to remove the *Nco*I site [35], and transformed into Stellar™ Competent Cells (Takara Bio USA, Inc.). Recombinant plasmid was confirmed by sequencing prior to recombination into BaculoDirect™ C-Term Linear DNA (Invitrogen) by LR Clonase™ II (Invitrogen). The recombinant BaculoDirect clone was transferred into Sf9 cells by transfection using Cellfectin II (Invitrogen) and amplified by successive infections of P1 and P2 viral stocks to a high-titer P3 viral stock. Protein expression was initiated by infecting 50 mL of 10^6^ cells/mL Sf9 cells in Sf-900 II SFM culture media supplemented with 10% (vol/vol) fetal bovine serum (Atlas Biologicals, Fort Collins, CO), 0.3 mM δ-aminolevulinic acid, and 0.1 mM ferric citrate with 50 μL of the P3 viral stock and incubating at 27 °C for 72 h. Recombinant CYP6DE3 and housefly cytochrome P450 reductase (HF-CPR) [2] were harvested 72 h post infection in a cell lysis buffer (100 mM sodium phosphate, pH 7.6, 20% (vol/vol) glycerol, 1.1 mM EDTA, 200 μM PMSF, and protease inhibitor cocktail (Sigma-Aldrich, St. Louis, MO) and the microsomes were isolated by differential centrifugation. The microsomal fraction was tested for functional CYP6DE3 using a CO-difference spectrum analysis [36].

### Enzyme assays

3-Carene, *R*-(+)-limonene, (+)-α-pinene and (*Z*)-dec-7-enal were obtained from Sigma-Aldrich. *cis*-3-Nonene was obtained from MP Biomedicals (Santa Ana, CA). Reaction mixtures consisted of 200 μL of the CYP6DE3 microsomal fraction, 40 μL of HF-CPR microsomal fraction, 100 mM sodium phosphate buffer pH 7.6, 1.5 mM NADPH (Sigma-Aldrich) and 21 mM of substrate in a total volume of 602 μL. Control reactions containing only HF-CPR were identical to the experimental reactions except that the reaction buffer was substituted for the CYP6DE3 microsomal fraction. Reactions were incubated in a capped 5 mL vial and rotated lengthwise at 30 °C in a FisherBiotech Hybridization Incubator (Thermo Fisher Scientific, Waltham, MA) for three hours. The reactions were terminated and extracted using pentane:ether (1:1). The extracts were analyzed by GC-MS on a HP-5 ms capillary column (Agilent) using an Agilent (Santa Clara, CA) 7890B gas chromatograph coupled to a 5977A mass spectrum detector. The instrument running parameters were: initial temperature of 40 °C with a one min hold, 5 °C/min to 240 °C and 15 °C/min to 300 °C with a 5 min hold. The MS detector was a single quadrapole with an electron ionization source and a molecular weight scanning range of 40 to 700 atomic mass units and an ionization potential of 70 eV.

### qRT-PCR of monoterpene exposed beetles

Beetles were separated by sex and placed in two oz plastic cups with perforated lids and incubated in a humidified dark drawer for 24 h, as described previously [3] to ensure they were unfed at the beginning of the monoterpene exposures. Small clumps of glass wool were placed in four pyrolized glass jars, two of which contained two mL vials capped with 500 μL of a selected monoterpene and a cotton mesh lid, or two jars with no vials as a control. Eight live males or females were transferred into each of the four jars so that each sex had monoterpene-exposed and control treatments. The jars were incubated in the dark for 24 h followed by placing 2-3 beetles in each of three replicate microcentrifuge tubes for each treatment and flash freezing them in liquid nitrogen. Six different monoterpenes were tested: 3-carene, *R*-(+)-limonene, myrcene, (+)-α-pinene, (–)-α-pinene, terpinolene. We also tested a monoterpene cocktail containing all six listed monoterpenes. RNA was extracted from the whole beetles using the RNeasy Plant Mini Kit from Qiagen as described above with an on-column DNase treatment. qRT-PCR was conducted using CYP6DE3 primers and normalized to YQE_05788 (rpS3P) as described above. Statistically significant differences between the means of relative expression between males and females for each gene were measured using an unpaired two-sample t-test at *p* < 0.05.

## Results

### RNA-Seq quality control and validation

**Fig. 2:**
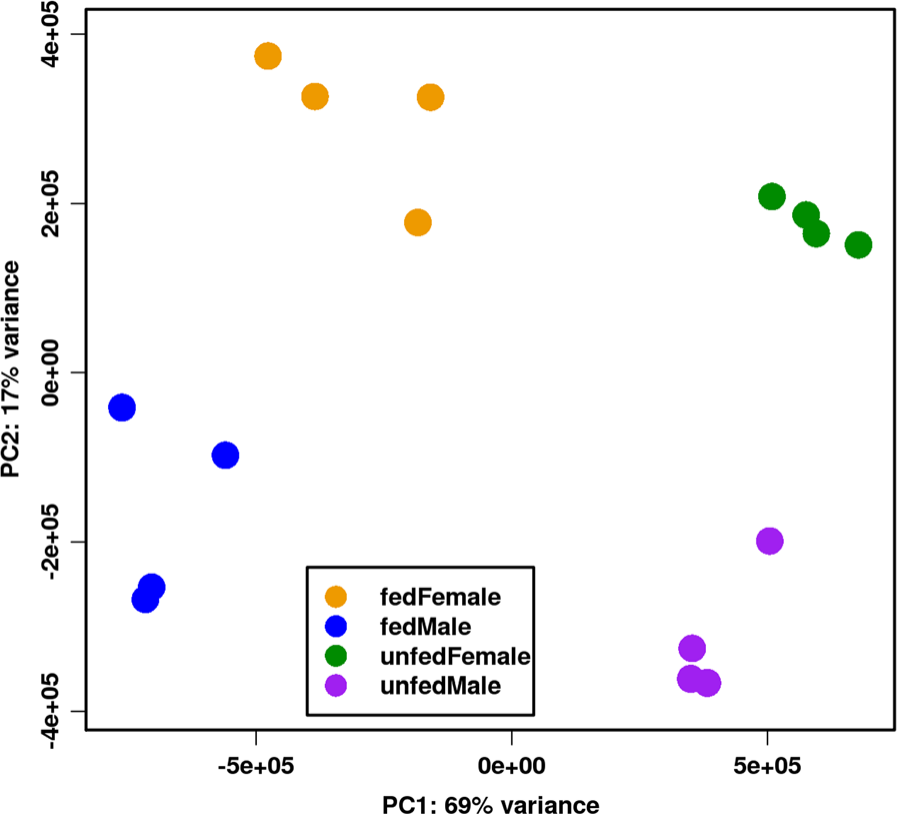
Principal Component Analysis. Principal component analysis of the filtered and normalized RNA-Seq expression levels of 11,342 transcripts shows a clear separation between feeding states. Almost 70% of the total variation is attributed to difference in feeding state

A total of 424,776,657 paired-end reads consisting of at least 76 bp were recovered from the 16 libraries, with reads per library ranging from 18,659,429 to 33,943,439 with a mean of 26,548,541. After processing, the number of reads aligned to the reference genome was 317,944,928 (Table 1), representing 13,407 potentially different transcripts. Of these transcripts a total of 11,342 transcripts passed the two filtering steps as explained in the Materials and Methods. Verification of RNA-Seq by qRT-PCR analysis confirmed similar expression measures between the two platforms. For the 15 genes in the seven samples measured by qRT-PCR and RNA-Seq, Pearson and Spearman Correlation Coefficients averaged to 0.924 and 0.878, respectively (Table 2). The PCA showed a clear separation between feeding states and genders, with almost 70% of the variance explained by feeding states (Fig. 2).

**Table 1:** Illumina NextSeq500 read processing and mapping results from RNA-seq mountain pine beetle libraries

Sample ID	Sample description	Biological replicate	Index 1	Index 2	Total read pairs	Processed read pairs	Aligned	Unique counts
MFF1	Adult fed female midgut/fat body	1	TCCGGAGA	AGGCTATA	32908461	32869809	25376605	20575844
MFF2	Adult fed female midgut/fat body	2	TCCGGAGA	GCCTCTAT	33943439	33904830	22563448	18078917
MFF3	Adult fed female midgut/fat body	3	TCCGGAGA	AGGATAGG	24984038	24959212	18972883	15336322
MFF4	Adult fed female midgut/fat body	4	TCCGGAGA	TCAGAGCC	32866187	32828407	21804076	17105387
MFM1	Adult fed male midgut/fat body	1	TCCGGAGA	CTTCGCCT	33881743	33839530	23694857	18773687
MFM2	Adult fed male midgut/fat body	2	TCCGGAGA	TAAGATTA	30640838	30616043	23809219	19162578
MFM3	Adult fed male midgut/fat body	3	TCCGGAGA	ACGTCCTG	29838624	29814207	21278225	17167448
MFM4	Adult fed male midgut/fat body	4	TCCGGAGA	GTCAGTAC	22832064	22820560	17562596	14236426
MUF1	Adult unfed female midgut/fat body	1	ATTACTCG	AGGCTATA	20441625	20422181	16173705	12413351
MUF2	Adult unfed female midgut/fat body	2	ATTACTCG	GCCTCTAT	21174699	21158515	17072234	12896359
MUF3	Adult unfed female midgut/fat body	3	ATTACTCG	AGGATAGG	20730531	20708211	16352452	12801594
MUF4	Adult unfed female midgut/fat body	4	ATTACTCG	TCAGAGCC	18659429	18650081	14552744	11262799
MUM1	Adult unfed male midgut/fat body	1	ATTACTCG	CTTCGCCT	24886800	24858754	19023568	14522591
MUM2	Adult unfed male midgut/fat body	2	ATTACTCG	TAAGATTA	28619496	28592550	22067707	16663228
MUM3	Adult unfed male midgut/fat body	3	ATTACTCG	ACGTCCTG	21795275	21769632	17025197	13146374
MUM4	Adult unfed male midgut/fat body	4	ATTACTCG	GTCAGTAC	26573408	26542803	20615412	15717687
	All libraries				424776657	424355325	317944928	249860592

**Table 2:** Correlation coefficients of RNA-Seq and qRT-PCR measured expression levels across seven samples and 15 genes

Gene	Pearson Corr.	Spearman Corr.
YQE_04793	0.966	0.964
YQE_11756	0.989	0.821
YQE_06376	0.992	0.927
YQE_05062	0.990	0.991
YQE_11643	0.931	0.929
YQE_09540	0.747	0.714
YQE_06803	0.923	1.000
YQE_06028	0.984	1.000
YQE_01079	0.802	0.607
YQE_06277	0.875	0.714
YQE_01901	0.925	0.893
YQE_02812	0.975	0.889
YQE_01868	0.994	0.991
YQE_01611	0.996	1.000
YQE_04799	0.945	0.893
AVERAGE	0.935	0.889

### Co-expression network

**petal** generated a co-expression network model based on Spearman Correlation Coefficient and similarity threshold of 0.808. This model includes 10,661 transcripts (94% of genes from the entire dataset) and follows the well-established biological network structure characteristics: small-world and scale-free [32]. From this model, closely connected gene groups based upon the genes of interest were extracted, resulting in thirteen vicinity networks (VNs). Here, a vicinity network (VN) is defined by the genes of interests and their common neighbors. For more detail refer to [32].

**Fig. 3:**
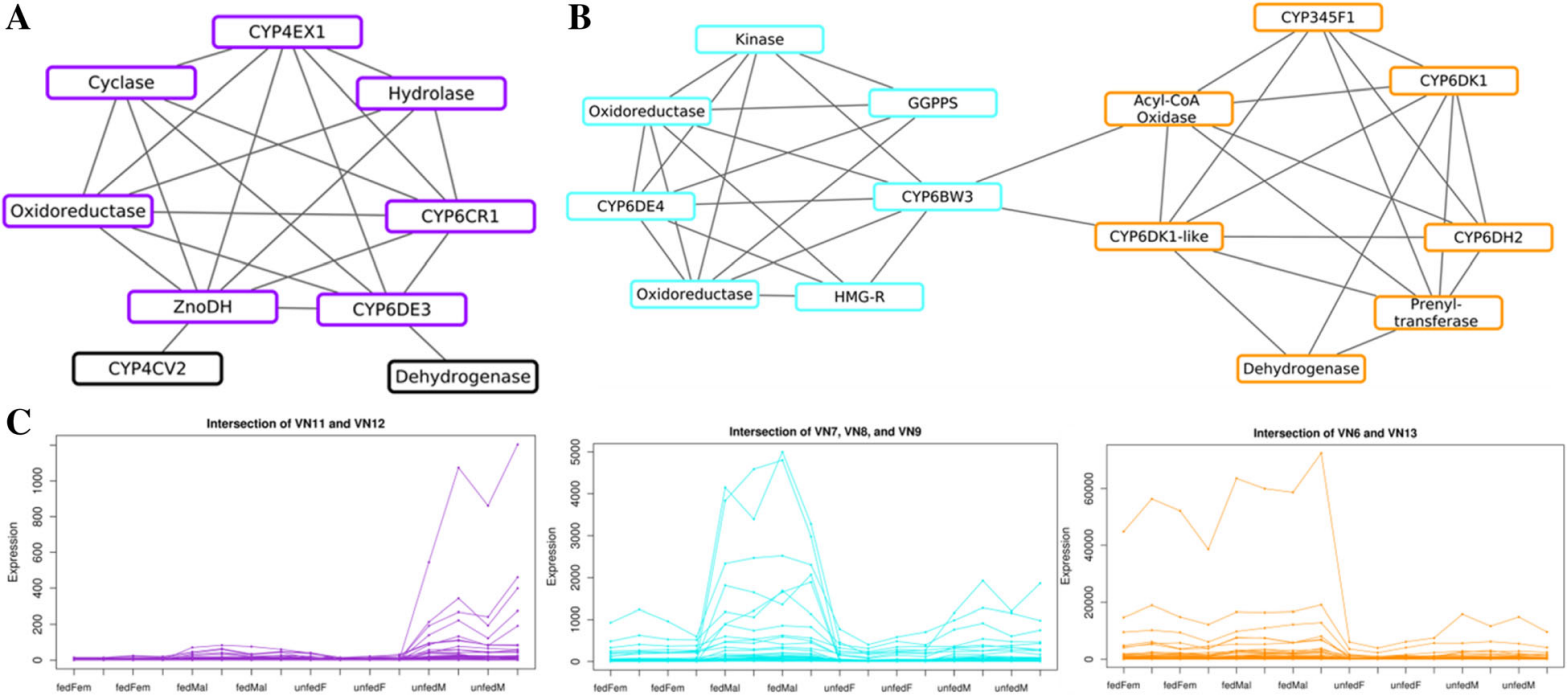
**petal** co-expression network analysis. Co-expression network model of a subnetwork of 23 genes hypothesized or confirmed to be involved in pheromone biosynthesis. Within the subnetwork, very densely connected groups, vicinity networks (VNs), are identified for (**a**) *exo*-brevicomin, highlighted *purple*, and (**b**) frontalin, highlighted *light blue* and *orange* (showing only the genes of interest.) The gene expression profiles of the intersections of VNs associated to the three colored gene groups are shown in (**c**) with a total of 31 genes in *purple*, 38 genes in *blue* and 84 genes in *orange* (see Additional file 3)

Based on the results from **petal**, further in-depth analyses were conducted leading to three distinct gene modules, one representing candidates involved in *exo*-brevicomin biosynthesis (purple), and two representing candidates involved in frontalin biosynthesis (light blue and orange) (Fig. 3a and b). Gene membership of each module is listed in Additional file 3.

### Gene ontology enrichment analysis of differential expressed genes

Based on the differential gene analysis 3,799 differentially expressed transcripts had a statistically significant (log_2_-fold change > 1) differential gene expression at a significance level of 0.01 after multiple testing adjustment (Table 3, Additional file 4). The greatest number of genes with a differential expression matching these criteria occurred in the MU > FU, MU > MF, and MF > MU comparisons with 894, 698, and 638 genes, respectively. The smallest numbers of genes with a differential expression matching our criteria were in the comparisons of FU > MU and FF > MF with 74 and 108 genes respectively.

**Table 3:** Summary of genes differentially expressed with greater than two fold change and adjusted *p*-value ≤ 0.01

Comparison	# of genes	Comparison	# of genes
Male Fed > Male Unfed	638	Male Unfed > Male Fed	698
Male Fed > Female Fed	446	Female Fed > Male Fed	108
Male Unfed > Female Unfed	894	Female Unfed > Male Unfed	74
Female Fed > Female Unfed	581	Female Unfed > Female Fed	360

**Fig. 4:**
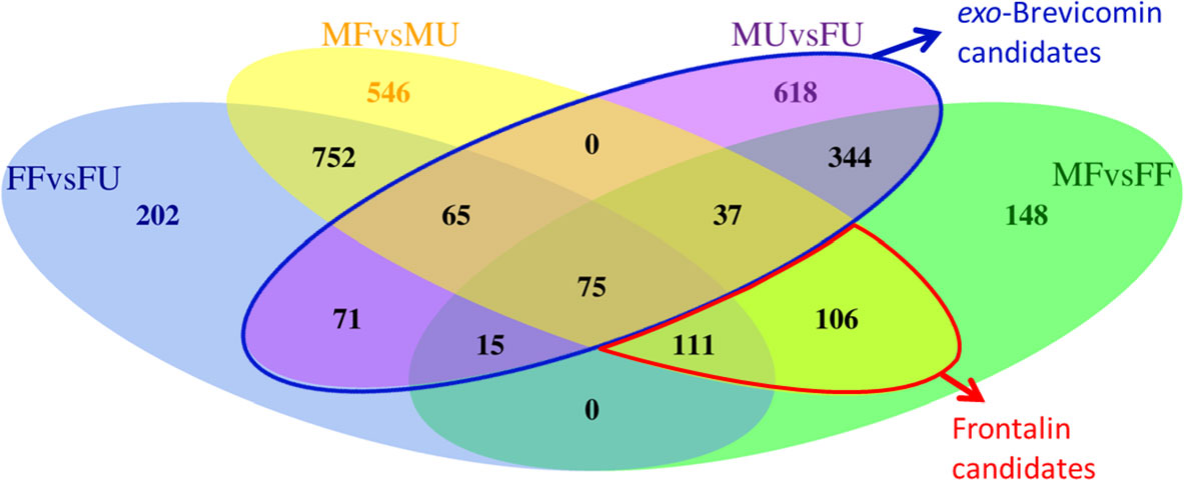
Venn diagram of over-expressed genes. A Venn diagram showing the intersections of statistically-significant differentially over-expressed genes (*p* < 0.01). The first treatment in each grouping has greater expression than the second treatment (i.e. FF > FU in the FFvsFU grouping). Two areas of interest included 1,422 genes: 1,225 genes as possible *exo*-brevicomin-biosynthetic genes (*blue outline*) and 217 genes as possible frontalin-biosynthetic genes (*red outline*)

Two areas highlighted in the Venn diagram (Fig. 4) were of particular interest as they represented pools of potential candidate genes for pheromone biosynthesis: 1) the 1225 genes with statistically significant differential expression between MU vs. FU beetles are *exo*-brevicomin-biosynthetic candidates; 2) the 217 genes with statistically significant differential expression in MF vs. MU and MF vs. FF, excluding differentially expressed genes in MU vs. FU, are potential frontalin-biosynthetic candidates.

**Fig. 5:**
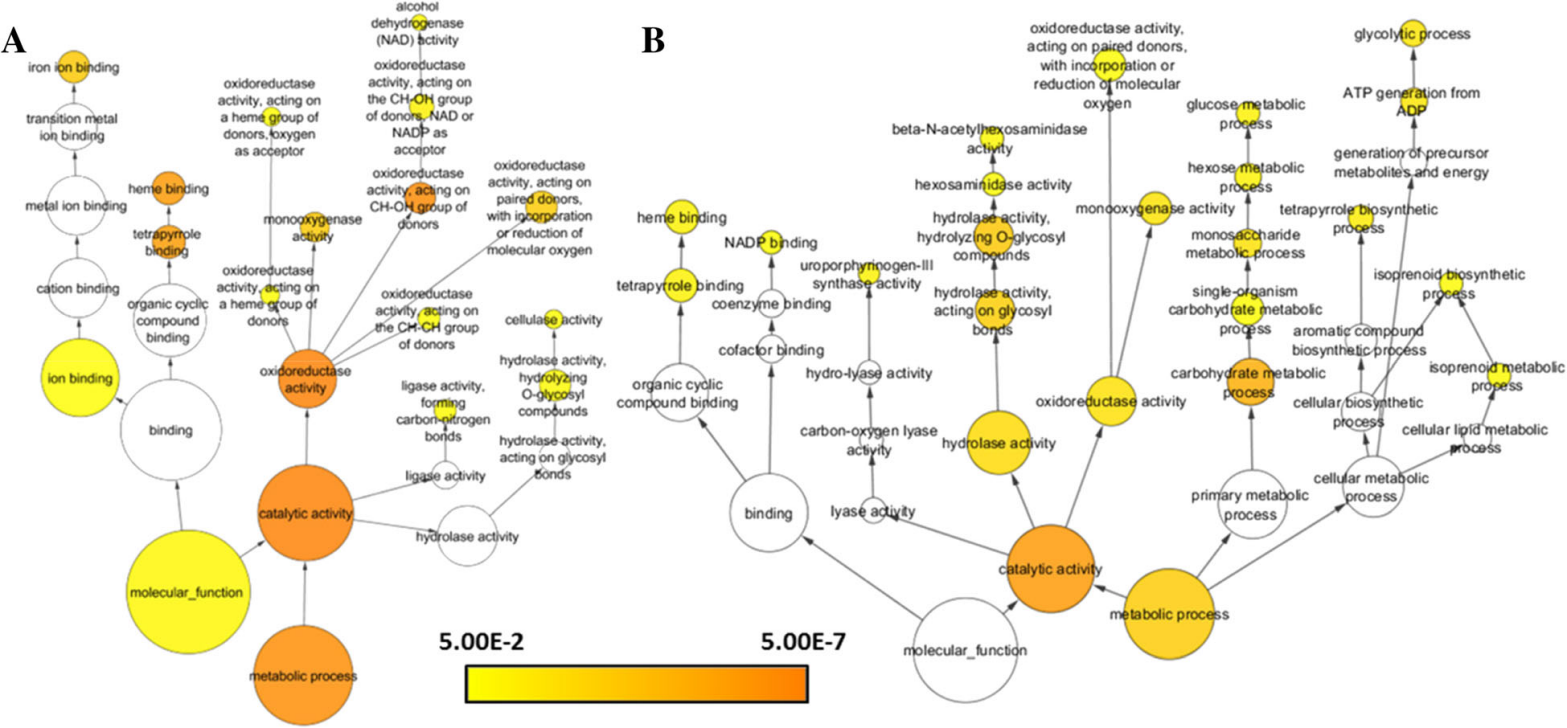
BiNGO analysis for candidate *exo*-brevicomin and frontalin biosynthetic genes. A BiNGO analysis showing gene ontology enrichment for **a** differentially over-expressed genes for MU > FU (possible *exo*-brevicomin-biosynthetic genes), *p* < 0.01, and **b** for both MF > MU and MF > FF (possible frontalin-biosynthetic genes), *p* < 0.01. Both comparisons show enrichment in P450 biosynthesis and oxidoreductase activity

To confirm potential candidate genes for pheromone biosynthesis, GO enrichment analysis was performed. Of the final 11,342 transcripts, 7,603 had at least one GO term. For the two pools of statistically significant differentially-expressed genes identified in the Venn diagram (possible *exo*-brevicomin- and frontalin-biosynthetic genes), a GO enrichment analysis using BiNGO within Cytoscape identified over-represented molecular functions and metabolic processes with an adjusted significance value of less than 0.05 (Fig. 5). Enriched GO terms in the possible *exo*-brevicomin-biosynthetic genes included P450-associated terms such as iron ion binding, heme binding and increased monooxygenase activity. Within the possible frontalin-biosynthetic genes, a wider variety of enriched metabolic processes are represented: uroporphyrinogen-III synthase and tetrapyrrole biosynthetic activity, monoxygenase activity, carbohydrate metabolic processes, isoprenoid biosynthetic processes, and two processes involved in P450 biosynthesis. Both gene groups showed enrichment in P450 related processes, however GO terms related to the mevalonate pathway were more highly enriched in the putative frontalin-synthesizing candidates.

### Pheromone biosynthesis candidate genes

#### Putative *trans*-verbenol-biosynthetic genes

**Fig. 6:**
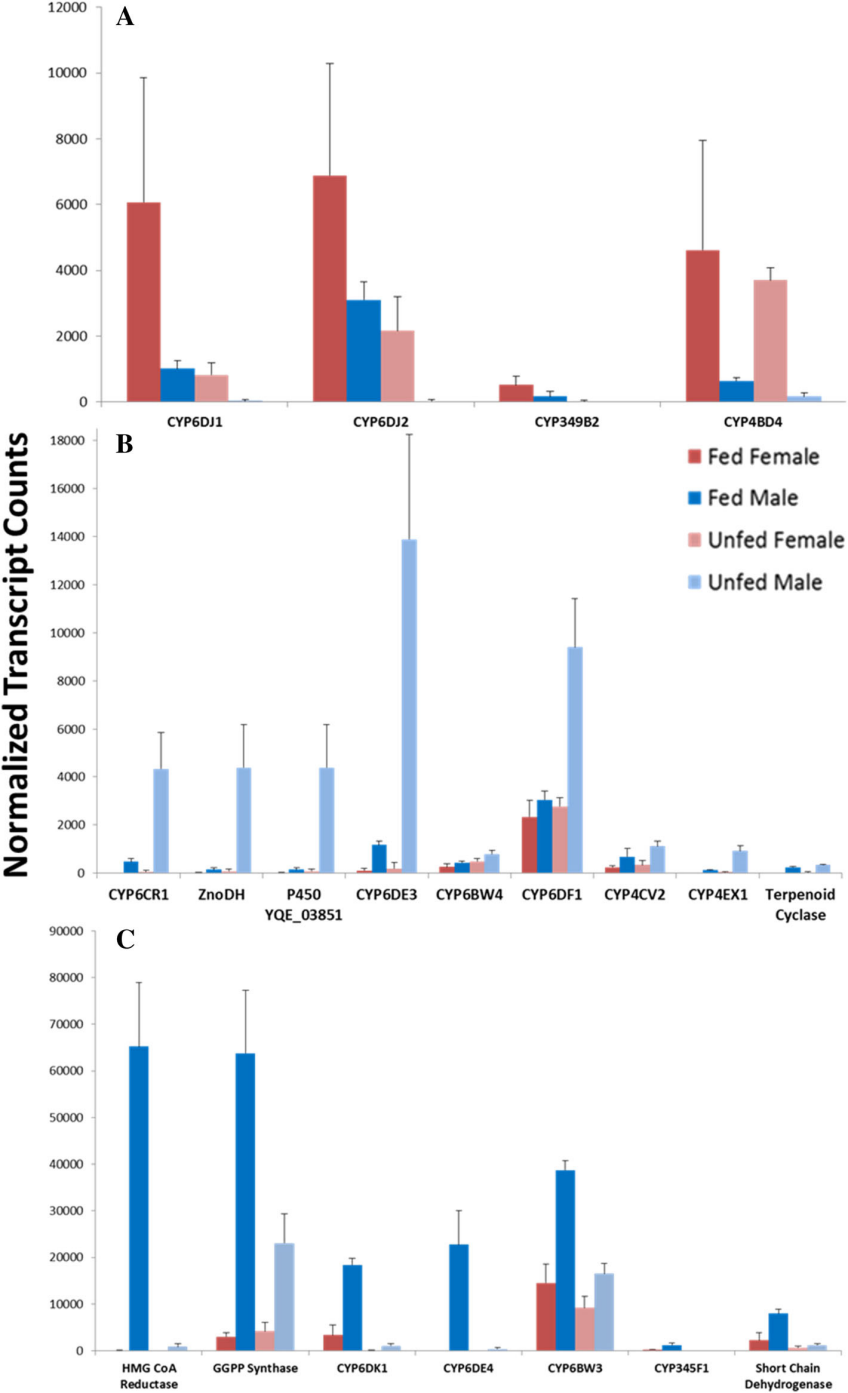
Candidate *trans*-verbenol, *exo*-brevicomin and frontalin biosynthetic genes. Expression profiles in regard to feeding status and sex for **a** four candidate genes hypothesized to be involved in *trans*-verbenol, **b** nine candidate genes hypothesized to be involved in *exo*-brevicomin and **c** seven candidate genes hypothesized to be involved in frontalin biosynthesis. Values represent the mean ± the standard deviation, *n* = 4

*trans-*Verbenol biosynthesis in female MPB likely takes place via P450-mediated hydroxylation of host (–)-α-pinene [37]. Three out of four P450 genes, *CYP6DJ1* (YQE_04799), *CYP6DJ2* (YQE_04800) and *CYP349B2* (YQE_02158) showed relatively high mRNA levels in fed females (Fig. 6a). One other P450, *CYP4BD4* (YQE_07200), showed mRNA levels in both fed and unfed females higher than those in males (Fig. 6a).

#### Putative *exo*-brevicomin-biosynthetic genes

*exo*-Brevicomin production from long chain fatty acid precursors in the fat body of unfed males [3] involves steps catalyzed by a desaturase, P450s, a short chain dehydrogenase, and a cyclase (Fig. 1c). The mRNA levels of two genes previously identified as active in the biosynthetic pathway, *CYP6CR1* and *ZnoDH* [2], were elevated in unfed males (Fig. 6b). Other P450 genes with high expression levels in unfed males included *CYP6DE3* (YQE_02812), *CYP6BW4* (YQE_01441), *CYP6DF1* (YQE_11788), *CYP4EX1* (YQE_01611), *CYP4CV2* (YQE_05823), a short chain dehydrogenase (YQE_04359) and one putative terpenoid cyclase (YQE_04789) (Fig. 6b). The expression profile of YQE_03851, which encodes a P450 with 98% a.a. identity to CYP4G56, similarly had increased expression levels in unfed males.

The purple gene module in Fig. 3a includes seven genes hypothesized or confirmed to be involved in *exo*-brevicomin biosynthesis (*CYP6CR1*, *ZnoDH*, *CYP6DE3*, *CYP4EX1*, a cyclase, an oxidoreductase and a hydrolase) and 22 common neighbors. This module is densely connected, with density equal to 0.987 and all genes having greater expression values in males than in females (Fig. 3c, Additional file 3). All gene members are statistically-significantly differentially-expressed (log_2_-fold change > 1) at a significance level of 0.01 (see Additional file 2).

#### Putative frontalin-biosynthetic genes

Steps for frontalin biosynthesis downstream of geranylgeranyl diphosphate are likely catalyzed by a dioxygenase or P450, and at least one additional P450 and a cyclase (Fig. 1b). Genes with higher relative expression in fed males were considered as candidates for frontalin biosynthesis. These included four P450s *CYP6DK1* (YQE_01078), *CYP6DE4* (YQE_01868), *CYP345F1* (YQE_06277) and *CYP6BW3* (YQE_02884) (Fig. 6c). No putative dioxygenase-encoding genes with elevated transcript levels in fed males compared to other treatments were identified. One putative short chain dehydrogenase, *YQE_11963*, and one putative terpenoid cyclase, *YQE_04789*, also had increased mRNA levels in fed males relative to the other groups (Fig. 6b and c).

The light blue gene module in Fig. 3b is based on seven genes previously hypothesized to play a role in frontalin biosynthesis: *CYP6DE4, CYP6BW3*, a kinase, two oxidoreductases, HMG-CoA reductase (*HMGR*), and geranylgeranyl diphosphate synthase (*GGPPS*). These seven genes and their 31 common neighbors created the densely connected light blue module (density = 0.983). The majority (26 of 38 genes) were statistically significantly over-expressed in fed males compared to unfed males and fed females (Fig. 3c).

Another interesting grouping is presented in the orange module. This module also includes seven putative candidate genes for frontalin biosynthesis: *CYP345F1, CYP6DK1, CYP6DH2,* a *CYP6DK1*-like P450, a prenyltransferase, an acyl-CoA oxidase, and a dehydrogenase (Fig. 3b). These seven candidate genes and their 77 common immediate neighbors are almost perfectly intra-connected (density = 0.994). Similar to the light blue module, the majority of genes (45) have a statistically significant higher expression (log_2_-fold change > 1) in fed male than unfed male and fed female (Fig. 3c).

### Functional analysis of CYP6DE3

**Fig. 7:**
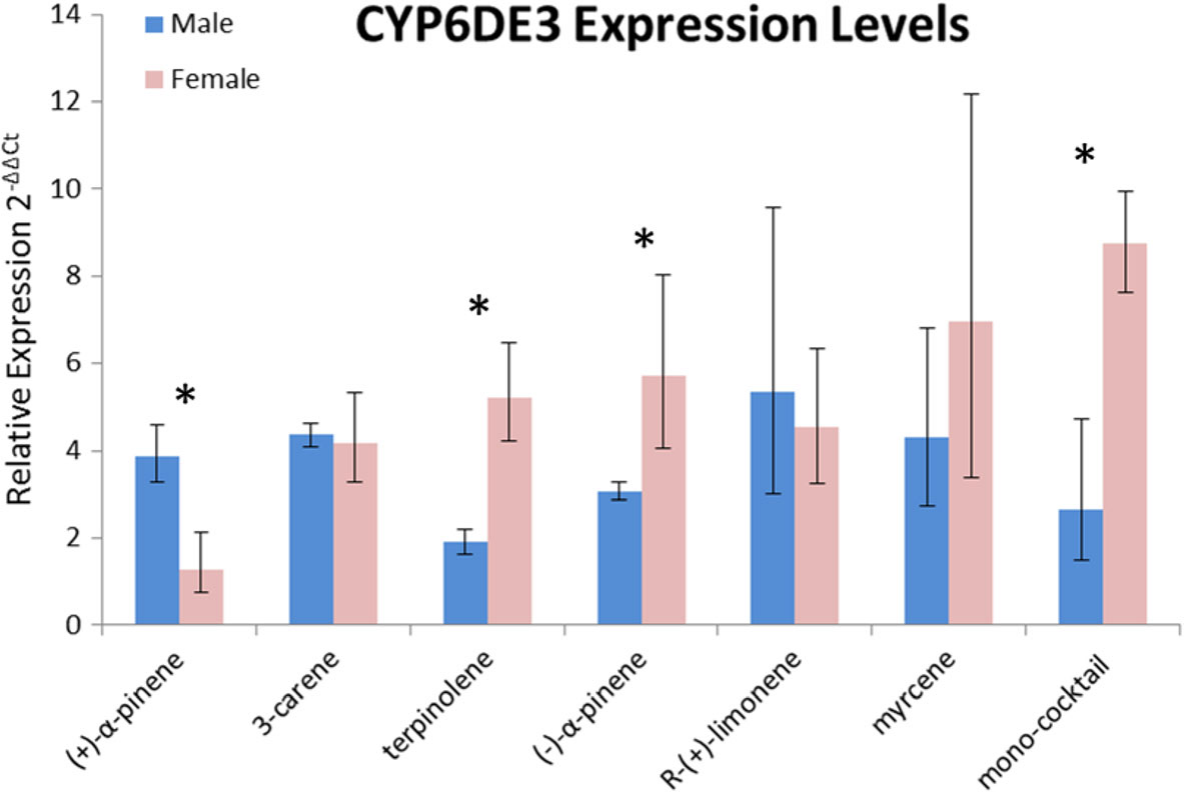
CYP6DE3 expression in response to monoterpene exposures. qRT-PCR analysis to measure relative CYP6DE3 mRNA levels in groups of adults (3 individuals per sample) exposed to atmosphere saturated with the indicated monoterpene for24 h. Normalized to RP S3, *n* = 3. Values represent the range of CYP6DE3 expression levels relative to the no exposure control. One asterisk indicates a statistically significant difference between the means of female ΔΔCt values compared to the means of male ΔΔCt values at *p* < 0.05

CYP6DE3 mRNA levels were elevated in all samples of unfed male and female beetles exposed to a variety of monoterpenes for 24 h compared to the controls. In general, monoterpene exposure elevated CYP6DE3 mRNA levels in females more strongly than in males, with the exception that (+)-α-pinene exposure elevated mRNA levels more strongly in males (Fig. 7).

**Fig. 8:**
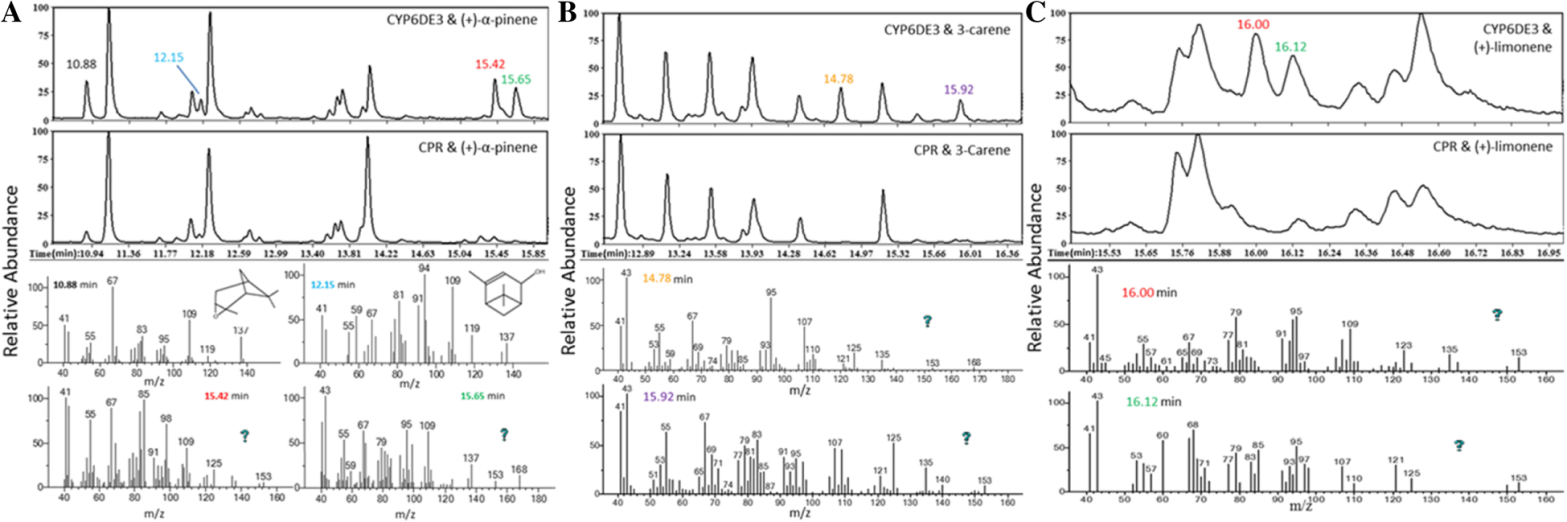
CYP6DE3 enzyme assays. GC chromatograms of pentane:ether (1:1) extracts from recombinant CYP6DE3 incubated for one hour in the presence of 6 mM **a** (+)-α-pinene, **b** 3-carene or **c** (+)-limonene, 3 mM NADPH and recombinant house fly CPR or with CPR alone (negative control). Below are mass spectra for selected retention peaks showing **a** production of 3-Oxatricyclo [4.1.1.0(2,4)]octane at 10.88 min, trans-verbenol at 12.15 min and two unknown products at 15.42 and 15.65 min for (+)-α-pinene, **b** two unknown products at 14.78 and 15.92 min for 3-carene and **c** to unknown products 16.00 and 16.12 for (+)-limonene. The 15.65 min product for (+)-α-pinene and the 14.78 min product for 3-carene have m/z peaks at 168 indicating a possible double oxidation of (+)-α-pinene and 3-carene

Recombinant CYP6DE3 was assayed for activity with hypothesized intermediates in the *exo*-brevicomin biosynthetic pathway, (Z)-dec-7-enal and (Z)-3-nonene (Fig. 1), as well as a variety of monoterpenes. Assays with (Z)-dec-7-enal or (Z)-3-nonene showed no unique products when analyzed by GC-MS compared to the negative controls, and neither were (Z)-3-nonene nor 6(Z)-nonen-2-ol, the hypothesized products, respectively, detected. The monoterpene substrates, however, showed unique or substantially increased GC-MS retention peaks compared to controls. Products resulting from (+)-α-pinene were tentatively identified as 3-oxatricyclo [4.1.1.0(2, 4)] octane (10.88 min) and *trans*-verbenol (12.15 min). Two other products (at 15.42 and 15.65 min) remain unidentified (Fig. 8a). Similarly, incubations with 3-carene as a substrate yielded two unknown products at 14.78 and 15.92 min (Fig. 8b), and incubations with (+)-limonene as a substrate also produced two unknown products (16.00 and 16.12 min; Fig. 8c).

## Discussion

Comparative transcriptome analysis to tentatively identify MPB genes encoding enzymes active in pheromone-biosynthetic pathways is based on the hypothesis that the genes for each pathway are coordinately regulated. It has proven useful for prior studies [15, 18]. We extended this approach here by comparing expression profiles in pheromone-biosynthetic tissues of fed and unfed female and male mountain pine beetles. Extensive RNA-Seq profiling yielded nearly 425 million paired-end reads, with 74.9% aligning to the reference genome. The clear separation of gender and feeding status shown by PCA underscores the remarkable shifts in genome usage exhibited by these beetles [7, 8]. The close correlation between RNA-Seq and qRT-PCR data (Table 2) supports that the expression values reported here reliably indicate in vivo mRNA levels.

We used a combination of bioinformatics analyses to narrow the pool of candidate pheromone-biosynthetic genes, beginning with four straight-forward comparisons of relative expression levels between physiological conditions. For example, *exo*-brevicomin-biosynthetic genes would be expected to have elevated expression levels in unfed males compared to both unfed females and fed males, whereas frontalin-biosynthetic genes would be higher in fed males compared to both unfed males and fed females. This produced preliminary pools of only ~200 – 1,200 candidates, depending on the analysis (Table 3; Fig. 3). These pools were enriched for P450 production and activity, consistent with increased metabolic activity upon feeding [7, 8, 18], which complicates identifying enzymes involved primarily in pheromone biosynthesis. Nevertheless, mevalonate pathway enzymes are more predominantly represented in fed males compared to females, consistent with frontalin production (Fig. 5). In parallel, co-expression network analysis by **petal** using a Spearman Correlation Coefficient and similarity threshold of 0.808 also isolated candidate genes. As these expression data were not normally distributed, the Spearman Correlation Coefficient supplied a robust non-parametric alternative to the standard Pearson Correlation Coefficient. Twenty-two of the final genes selected by the **petal** analysis appear relevant to *exo*-brevicomin biosynthesis, while another 71 may be involved in frontalin production.

(–)-*trans*-Verbenol is produced by a single P450-mediated hydroxylation of (–)-α-pinene, a reaction that may be catalyzed by multiple enzymes as part of a detoxification process [37]. Thus, a “pheromone-biosynthetic” P450 that specifically produces *trans*-verbenol in females may be an artificial designation. Our current study notes three P450s with relatively high expression levels in fed females compared to unfed females and males (Fig. 6a). A fourth P450, *CYP4BD4*, showed the highest mRNA levels in females compared to males, though in a pattern that is not consistent with feeding-induced *trans*-verbenol production.

*exo*-Brevicomin production from long chain fatty acid precursors in the fat body of unfed males [3] involves steps catalyzed by P450s, a short chain dehydrogenase, and a cyclase (Fig. 1c). High probability candidate genes for *exo*-brevicomin biosynthesis are likely in the same **petal** group containing *CYP6CR1* and *ZnoDH* (Fig. 3a). Interestingly, this gene group includes a putative cyclase (*YQE_04789*) that may catalyze the terminal reaction. The two P450s (*CYP6DE3* and *CYP4EX1*) may be active upstream of *ZnoDH* to produce and/or hydroxylate 3-nonene. In this respect, the CYP4G56-like P450 (*YQE_03851*) was not part of the gene group but is of interest given its similar expression profile (Fig. 6b) and identity as a CYP4G. While predicting P450 function from sequences is very difficult, CYP4G family P450s appear to be insect-specific and function as oxidative decarbonylases – yielding hydrocarbons from long chain fatty aldehydes [38, 39]. Thus, *YQE_03851* may contribute to 3-nonene production.

Frontalin-biosynthetic steps through the mevalonate pathway to geranylgeranyl diphosphate are well established in fed and JH treated MPB males [40]. Our analysis also identified mRNAs for mevalonate pathway enzymes, including HMGR and GGPPS, to be elevated in fed males compared to other treatment groups. Later steps are likely catalyzed by P450s, a dioxygenase, and a cyclase that should group together with HMGR and GGPPS in the **petal** analysis. Two P450 genes, CYP6DE4 (*YQE_01868*) and CYP6BW3 (*YQE_02884*), did group with HMGR and GGPS (light blue VN in Fig. 3b) while four other P450 genes, CYP345F1 (*YQE_06277*), CYP6DK1 (*YQE_01078*), CYP6DH2 (*YQE_01329*) and a CYP6DK1-like P450 (*YQE_01079*), grouped into one different VN (orange VN in Fig. 3b). The two VNs are connected directly by two links, and both gene groups portray increased expression in fed males (Fig. 3c), a pattern consistent with frontalin biosynthesis. Interestingly, a putative dioxygenase was not identified, which may suggest alternative activities on a GGPP precursor, perhaps catalyzed by a cytochrome P450. It is also noteworthy that the cyclase identified in the “*exo-*brevicomin cluster” (YQE_04789) also shows elevated mRNA levels in fed males (Fig. 6b). Given the structural similarities of the epoxide precursors for both *exo*-brevicomin and frontalin, it is possible that a single cyclase could serve the terminal steps in both pathways.

While comparative transcriptomics is invaluable to preliminarily identify putative pheromone-biosynthetic genes, a more accurate assessment requires additional information [10]. For MPB, our transcriptomic analyses return more candidate genes than there are reactions to catalyze. We hypothesized that those with elevated expression upon exposure to monoterpenes are more likely to contribute to resin detoxification than pheromone component production (except for the case of *trans*-verbenol, as noted above). We therefore measured relative mRNA levels for *CYP6DE3*, which we had tagged as a potential *exo*-brevicomin biosynthetic enzyme, in beetles that had been exposed to atmospheres saturated with various monoterpenes. The clear elevation observed for all cases (Fig. 7) suggests that *CYP6DE3* is induced by monoterpene exposure, particularly in females, implying a resin-detoxifying role. The absence of this induction in fed insects further implicates that *CYP6DE3* regulation is complex. The monoterpene-dependent difference in response in males and females is curious, but has been exhibited in another study reporting similar sex-specific transcriptional responses of various *D. armandi* P450 genes in response to monoterpenes [41]. A detoxification role for CYP6DE3 is supported by functional assays of the recombinant enzyme which showed that it oxidized a variety of monoterpenes, but did not appear to accept *exo*-brevicomin precursors as substrates (Fig. 8 and data not shown). Interestingly, the products at 15.65 min for (+)-α-pinene and 14.78 min for 3-carene have a m/z peak at 168 suggesting these substrates were oxidized twice (Fig. 8a and b).

De novo pheromone component biosynthesis in pine bark beetles is affected by sex, feeding status, environment, and JH III [10], with JH III treatment sometimes being sufficient to elevate mRNA levels of pheromone-biosynthetic genes even in insects that otherwise require feeding to trigger pheromone production [17]. Indeed, JH III stimulates both frontalin [5, 11] and *trans-*verbenol biosynthesis, but not *exo*-brevicomin biosynthesis [11] in MPB. Our study complements those of Robert et al. [8], who compared fed and JH III-treated whole insects and concentrated on a survey of detoxification mechanisms, and Keeling et al. [11], who compared starved and JH III-treated midguts and fat bodies. Our study differs in that we focused on midgut and fat body tissues of fed and unfed insects rather than JH III-treated insects because of the evident complexity in regulating production of these three main pheromone components. Several putative pheromone-biosynthetic genes identified in our study agree with those reported by Keeling et al. [11] (Table 4), and the increased confidence resulting from this concurrence makes the common genes high priorities for functional assays. It is also noteworthy that CYP6DE4 does not accept pheromone precursors despite being induced by JH III [11]. The discrepancies in the list of candidate enzymes are likely due to a combination of factors, including differences in experimental design and data analysis. Given that the populations used by Keeling et al. [11] and us appear to be geographically and genetically isolated [42], it is also possible that their responses to different conditions also differ ([2], unpublished data).

**Table 4:** Candidate genes for MPB pheromone biosynthesis identified by RNA-seq using feeding status or JH treatment

	Feeding status and sex	JH treatment and sex (Keeling et al. [11])	Proposed in both studies
Pheromone biosynthetic pathway			
*trans*-Verbenol	CYP349B2		CYP6DJ1
			CYP6DJ2
*exo*-Brevicomin	CYP6DE3	CYP6DE4	CYP4EX1
	CYP6BW4	CYP18A1	CYP6DF1
	CYP4CV2	CYP4BQ1	CYP6CR1
	P450 YQE_03851		ZnoDH
	Cyclase YQE_04789		
Frontalin	CYP6DE4	CYP6BW1	HMGR YQE_02503
	CYP6BW3		GGPPS YQE_09494
	CYP345F1		CYP6DK1
	Cyclase YQE_04789		

## Conclusions

This study identified a number of candidate genes for involvement in MPB aggregation and anti-aggregation pheromone biosynthesis through differential gene expression analysis based on sex and feeding status. However, as Keeling et al. [11] noted, caution should be employed when using comparative transcriptomic data to identify putative pheromone-biosynthetic genes. As evidenced by the functional analysis of an identified candidate gene, CYP6DE3, for *exo*-brevicomin biosynthesis in MPB, expression profiles are not always indicative of a specific role for a gene of interest. Further functional analysis of the genes identified in this study should lead to the discovery of most, if not all, of the unknown enzymes involved in MPB aggregation and anti-aggregation pheromone biosynthesis.

## Additional files

Additional file 1: Primer Table: A list of primers used for qRT-PCR and CYP6DE3 cloning. (XLSX 10 kb)

Additional file 2: **petal** Venn Diagrams: Venn diagrams showing the intersections of differentially over-expressed genes with a < 0.01 for the (A) purple, (B) light blue and (C) orange gene groups identified in the **petal** analysis. “gr” indicates greater than in each of the comparisons. (PPTX 178 kb)

Additional file 3: **petal** Module Gene Annotations: A list of genes and their annotations found in each **petal** module. (XLSX 20 kb)

Additional file 4: Differentially Expressed Genes for Each Comparison: A list of all the genes that have greater than two fold differential expression for each considered comparison of sex and feeding status. (XLSX 1704 kb)

## Abbreviations

a.a.: Amino acid
bp: Base pair
cDNA: Complementary deoxyribonucleic acid
EDTA: Ethylenediaminetetraacetic acid
FF: Female fed
FU: Female unfed
GC-MS: Gas chromatography-mass spectrometry
GGF: Georgia Genomics Facility
GGPPS: Geranygeranyl diphosphate synthase
GO: Gene ontology
GTF: Gene transfer format
HF-CPR: House fly cytochrome P450 reductase
HMGR: 3-hydroxy-3-methylglutaryl-CoA reductase
JH: Juvenile hormone
MF: Male fed
MPB: Mountain pine beetle
mRNA: Messenger ribonucleic acid
MU: Male unfed
NADPH: Nicotinamide adenine dinucleotide phosphate
nt: Nucleotide
ORF: Open reading frame
P450: Cytochrome P450 monooxygenase
PCA: Principal component analysis
PCR: Polymerase chain reaction
PMSF: Phenylmethylsulfonyl fluoride
qRT-PCR: Quantitative reverse transcriptase PCR
RNA-Seq: RNA sequencing
SAM: Sequence alignment/map
VN: Vicinity network
