# Supplementary material for: Comparative transcriptomics of mountain pine beetle pheromone-biosynthetic tissues and functional analysis of CYP6DE3
Source: BMC Genomics. 2017 Apr 20;18:311. doi: 10.1186/s12864-017-3696-4 (PMC5397757; doi:10.1186/s12864-017-3696-4)
Supplement: Supplementary file 2 — petal Venn Diagrams: Venn diagrams showing the intersections of differentially over-expressed genes with a < 0.01 for the (A) purple, (B) light blue and (C) orange gene groups identified in the petal analysis. “gr” indicates greater than in each of the comparisons. (PPTX 178 kb) [file 12864_2017_3696_MOESM2_ESM.pptx]

## Slide 1
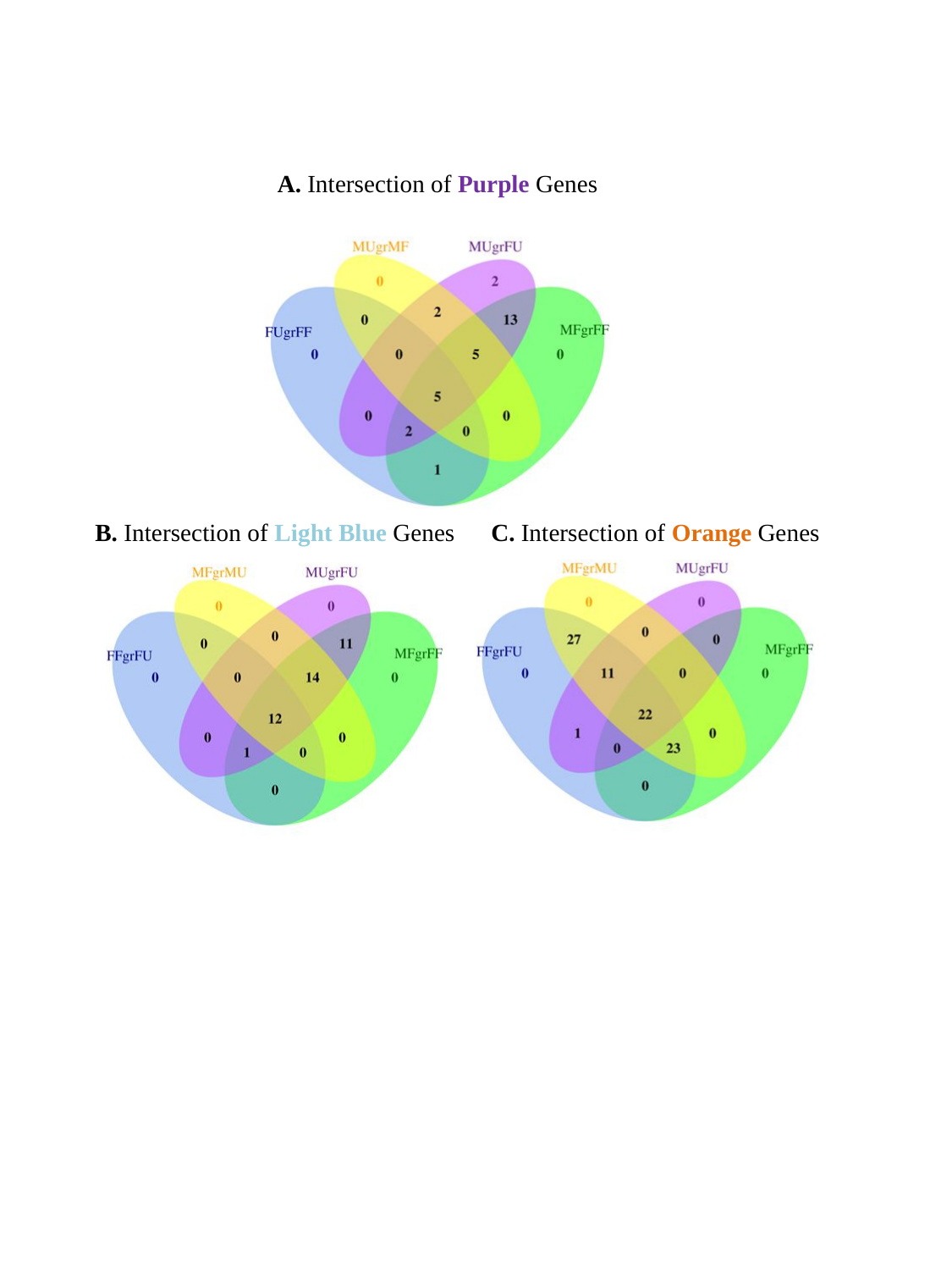

A. Intersection of Purple Genes
B. Intersection of Light Blue Genes
C. Intersection of Orange Genes
